# Supplementary material for: Transgenic HA-1-Specific CD8+ T-Lymphocytes Selectively Target Leukemic Cells
Source: Cancers (Basel). 2023 Mar 3;15(5):1592. doi: 10.3390/cancers15051592 (PMC10000933; doi:10.3390/cancers15051592)
Supplement: Supplementary file 1 [file cancers-15-01592-s001.zip › Supplementary materials/supplementary materials.pdf]

Transgenic HA-1-specific CD8^{+} T-lymphocytes selectively target leukemic cells.
A. Pilunov *et al*

Supplementary methods

Flow cytometry analysis

T cell expansions were analyzed for the HA-1 specificity using in-house manufactured HA-1 HLA-A*02.01 MHC-tetramers according to the previously published protocol (30). Briefly, 200 ng of biotin-conjugated HLA-A*02.01 MHC monomers were mixed with 200 ng of streptavidin-phycoerythrin or streptavidin-allophycocyanin conjugates (Thermo Fisher Scientific, USA) in 10 µl of phosphate buffer saline (PBS) containing 0.5 % BSA and 2mM EDTA for 45 minutes on ice in the dark. Assembled MHC-tetramers were added to the T-cells, resuspended in 20 µl of IS buffer (IBA Biosciences, Germany) and incubated for 30 minutes at 37°C in the dark. For further flow cytometry analysis cell samples were then stained with antibodies in 50 µl of PBS buffer for 15 minutes at 37°C.

For the analysis of endogenous TCR knockout efficiency, the cells recovered for 72h after electroporation (see methods, section “CRISPR/Cas knockout of endogenous TCR”) and were stained with anti-human TCR and anti-CD3 antibodies (suppl. table 1). The knockout efficiency was calculated as percentage of the TCR^{-} CD3^{-} cells in the population.

Flow cytometry analysis was performed on FACSCanto™ II (BD Biosciences, USA) with further data analysis using FlowJo 10 software (BD Biosciences, USA). The panel of antibodies used for the flow cytometry analysis is present in supplementary table 1.

LCL generation

To create lymphoblastoid cell line (B-LCL), Epstein-Barr virus particles produced from B95-8 (ATCC VR-1492™) cell line were used. $3*10^6$ PBMC from healthy donors were resuspended in 3 ml of medium obtained after 3-day culturing of B95-8 (RPMI with 20% FBS and 1% of 10000 U/ml penicillin/streptomycin). After 24 hours of incubation at 37°C in a CO2 incubator, 3 ml of RPMI medium containing and 200 ng/mL of cyclosporine A was added to PBMC culture. Two weeks later the cells were transferred to a medium containing 10% FBS.

Lentivirus manufacturing

For lentivirus manufacturing, HEK293T (ATCC CRL-3216^{TM}) cells were cultured in DMEM (Gibco, UK) supplemented with 10% FBS and 1% of 10000 U/ml penicillin/streptomycin. The lentiviral plasmid containing assembled transgenic TCR was co-transfected with packaging plasmids (ViraPower packaging system, Thermo Fisher Scientific, USA) into HEK293T cell line using lipofectamine 2000 (Thermo Fisher Scientific, USA) according to the manufacturer's protocol. Cells were cultivated for 72 h after transfection, the supernatant was collected, purified by 0.45 µm filtration and concentrated using Lenti-X concentrator (Takara, Japan). The pellet was reconstituted in the RPMI medium, aliquoted, and stored at -80 °C. The lentiviral transduction units (TU) were determined by titration of the J76 cell line with different virus dilutions. The TCR expression was assessed by flow cytometry analysis of CD3 expression. The average titer in obtained viral preparations was $2\text{-}5*10^7$ TU/ml after concentration.

Lentiviral transduction of cell lines

Transduction of J76 and K562 cell lines was carried out as followed. Cells (10^6 cells/well) were seeded in the 24-well plate overnight. Concentrated lentivirus preparations were added to achieve (MOI)~0.2 in case of TCR-expressing J76 cells to ensure that the majority of transgene-positive cells contains no more than one

viral integration per cell, according to Poisson's distribution. Transduced cells were enriched after 72 h by immunomagnetic separation for CD3 (Miltenyi Biotec, Germany). Transduction efficiency was assessed by flow cytometry analysis of CD3 expression and cells were purified using BD FACS Aria III.

Ribonucleoprotein production

The required gRNA was synthesized *in vitro* with TranscriptAid T7 High Yield Transcription Kit (Thermo, USA) from a DNA template prepared by overlap-extension PCR. Synthesized gRNA was then treated with CIP phosphatase (NEB, USA) and purified on RNEasy mini kit columns (Qiagen, Germany) according to the published protocol (37). Both previously reported gRNA sequences (38,39) and a set of newly designed gRNAs by Synthego (www.synthego.com) we prepared as above.

Approximately 90 pmol of gRNA were combined with 30 pmol of True Cut V2 Cas9 protein (Thermo Scientific, USA) in the 3:1 gRNA to Cas9 molar ratio per one transfection. Buffer T (Neon electroporation kit) was added up to 20 µl and mix was incubated for 20 min at room temperature. Obtained RNP complexes were used directly for electroporation of isolated $CD8^+$ T cells.

Supplementary table 1. Flow cytometry reagents

Antibody/staining agent	Fluorophore	Manufacturer
anti-human HLA-A2, Mouse IgG2b, κ, Clone BB7.2	PE	BD Biosciences, USA
anti-CD137, Mouse IgG1, κ, Clone 4B4-1	PE	Miltenyi Biotec, Germany
anti-human TCR α/β, Mouse IgG1, κ, Clone IP26	APC	Sony, Japan
anti-CD3, Mouse, IgG1, κ, Clone UCHT1	AF700	Sony, Japan
anti-mouse TCR β, Hamster IgG2, λ1, Clone H57-597	APC	BD Biosciences, USA
HA-1-HLA Dextramer	PE	Immudex, Denmark

Supplementary table 2. T cell expansions summary data

Donor	HLA-A02 genotype of CD8+ T cells donor	Expansion typed	Wells with antigen-specific clones/wells in total	Antigen-specific fraction obtaining method	Chains cloned/enriched	TCRs functional/tested
P005	+	Autologous	10/35	Separation of Tetramer^{+} fraction	11/22 α<br>10/12 β	4/20
P845	+	Autologous	15/22	Separation of CD137^{+} fraction after restimulation	8/8 α<br>6/11 β	4/9
P1102	+	Autologous	6/22	Separation of Tetramer^{+} fraction	7/11 α<br>6/6 β	3/15
P180	+	Autologous	3/15	Separation of CD137^{+} fraction after restimulation	No α chains sequenced<br>0/17	N/A
P1035	+	Autologous	5/15	Separation of Tetramer^{+} fraction	No α chains sequenced<br>0/6	N/A
P196	-	Allogeneic	3/6	Separation of Tetramer^{+} fraction	0/3 α<br>0/3 β	N/A
P1329	-	Allogeneic	3/12	Separation of Tetramer^{+} fraction	2/5 α<br>2/4 β	2/2
P856	-	Allogeneic	1/6	Separation of Tetramer^{+} fraction	0/1 α<br>0/1 β	N/A

Supplementary table 5.

Number of donors with HLA alleles tested for the alloreactivity.

HLA	Donors, n	HLA	Donors, n	HLA	Donors, n
A*01	14	B*07	3	C*02	2
A*03	3	B*08	8	C*03	5
A*11	1	B*13	1	C*04	3
A*24	5	B*14	1	C*05	1
A*25	2	B*15	1	C*06	2
A*26	3	B*27	2	C*07	9
A*31	1	B*35	5	C*08	1
A*32	1	B*37	2	C*12	3
A*33	2	B*39	3	C*14	1
A*68	1	B*40	4	C*16	2
		B*41	2	C*17	1
		B*49	1		
		B*44	2		
		B*51	1		
		B*52	1		
		B*57	2		

Supplementary table 6.

TRAC- and TRBC-specific guide RNAs sequences and their knockout efficiencies in CD8+ T cells.

**Name**	**Gene**	**Sequence**	**Source**	**Knockout efficiency**
gRNAa1	TRAC	AGAGTCTCTCAGCTGGTACA	(53)	86%
gRNAa2	TRAC	GCUGGUACACGGCAGGGUCA	Synthego	37%
gRNAa3	TRAC	CUCUCAGCUGGUACACGGCA	Synthego	7%
gRNAa4	TRAC	UCAGGGUUCTGGAUAUCUGU	(52)	53%
gRNAa5	TRAC	UCUCUCAGCUGGUACACGGC	Synthego	11%
gRNAb1	TRBC1/2	CUUUCCAGAGGACCUGAACA	Synthego	6%
gRNAb2	TRBC1/2	CAGGGAAGAAGCCUGUGGCC	Synthego	2%
gRNAb3	TRBC1/2	GUGGUCAGGGAAGAAGCCUG	Synthego	45%
gRNAb4	TRBC1/2	AGGCUUCUUCCCUGACCACG	Synthego	4%
gRNAb5	TRBC1/2	CAAACACAGCGACCTTGGGT	(53)	57%
gRNAb6	TRBC1/2	AACACGUUUUUCAGGUCCUC	Synthego	14%
gRNAb7	TRBC1/2	CGGGGUAGAAGCCUGUGGCC	Synthego	7%
gRNAb8	TRBC1/2	AGAGAUCUCCCACACCCAAA	(52)	36%

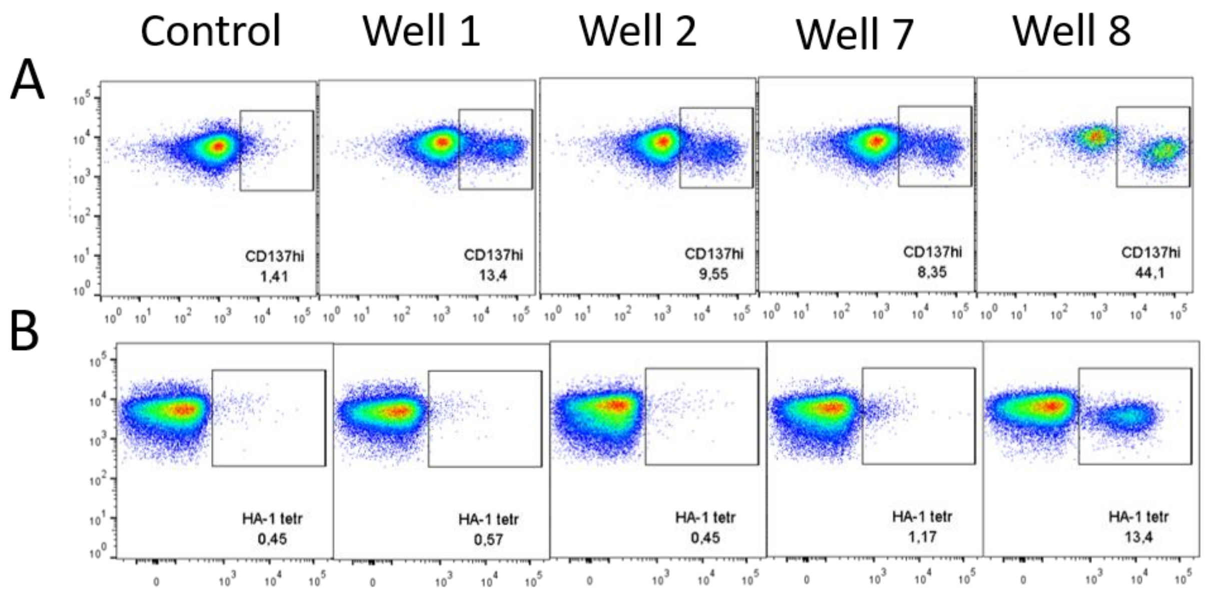

Supplementary figure 1. Antigen-specific expansion wells demonstrate tetramer and CD137 staining. Representative plots of CD137 (A) and tetramer staining (B) **for** expansions of clones obtained from donor p845.

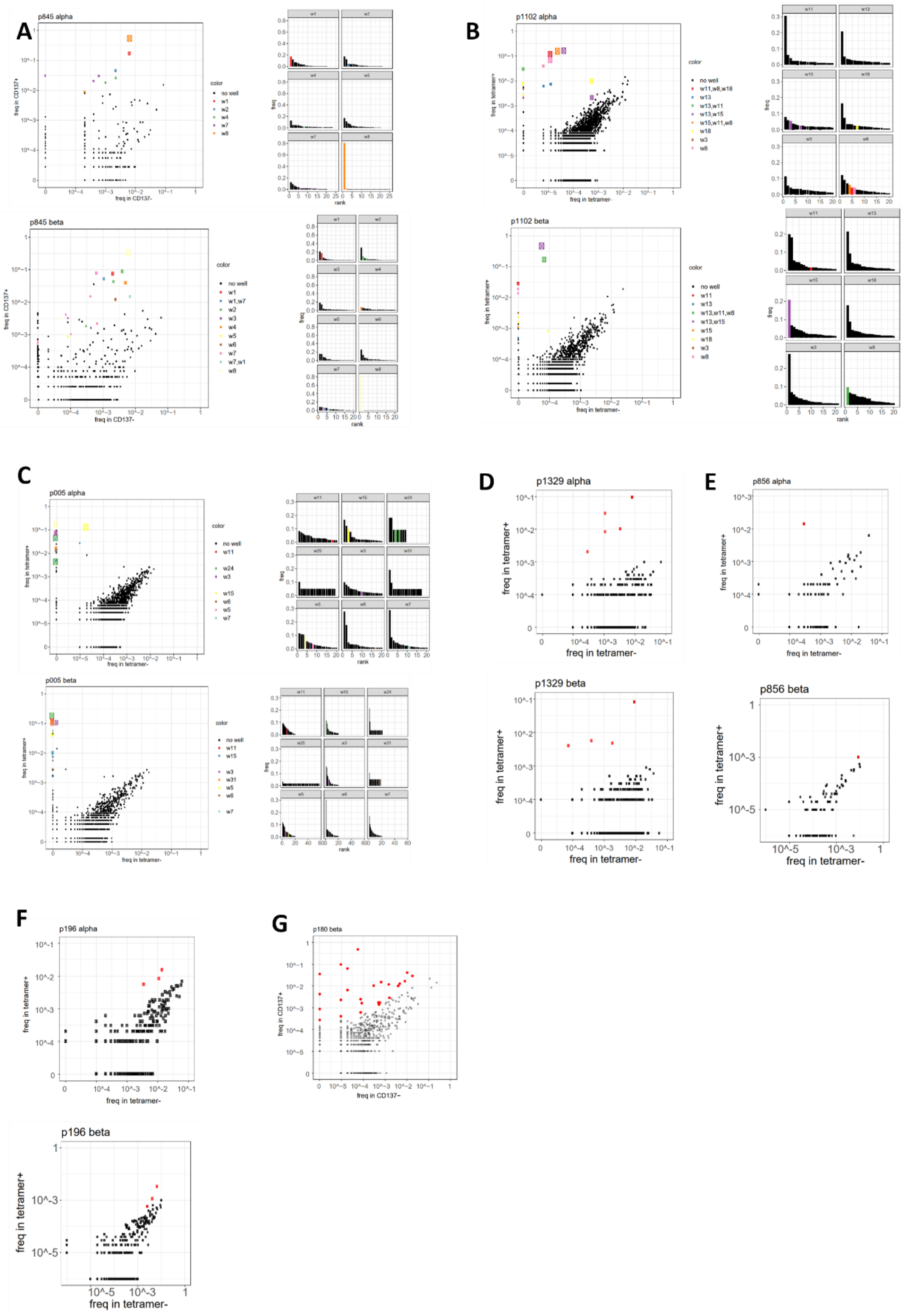

Supplementary figure 2. **Enrichment plots of HA-1-specific expansions.** Frequency of the TCR reads in flowthrough (X axis) and antigen-specific fraction (Y axis). Each dot represents a unique TCR chain, antigen-specific chains are colored. **A, B, C.** Enrichment plots for donors p845, p1102 and p005 respectively. Frequency ranks of enriched chains are shown to the right of the enrichment plots in each expansion well. **D, E, F, G.** enrichment plots for donors p1329, p856, p196 and p180 respectively.

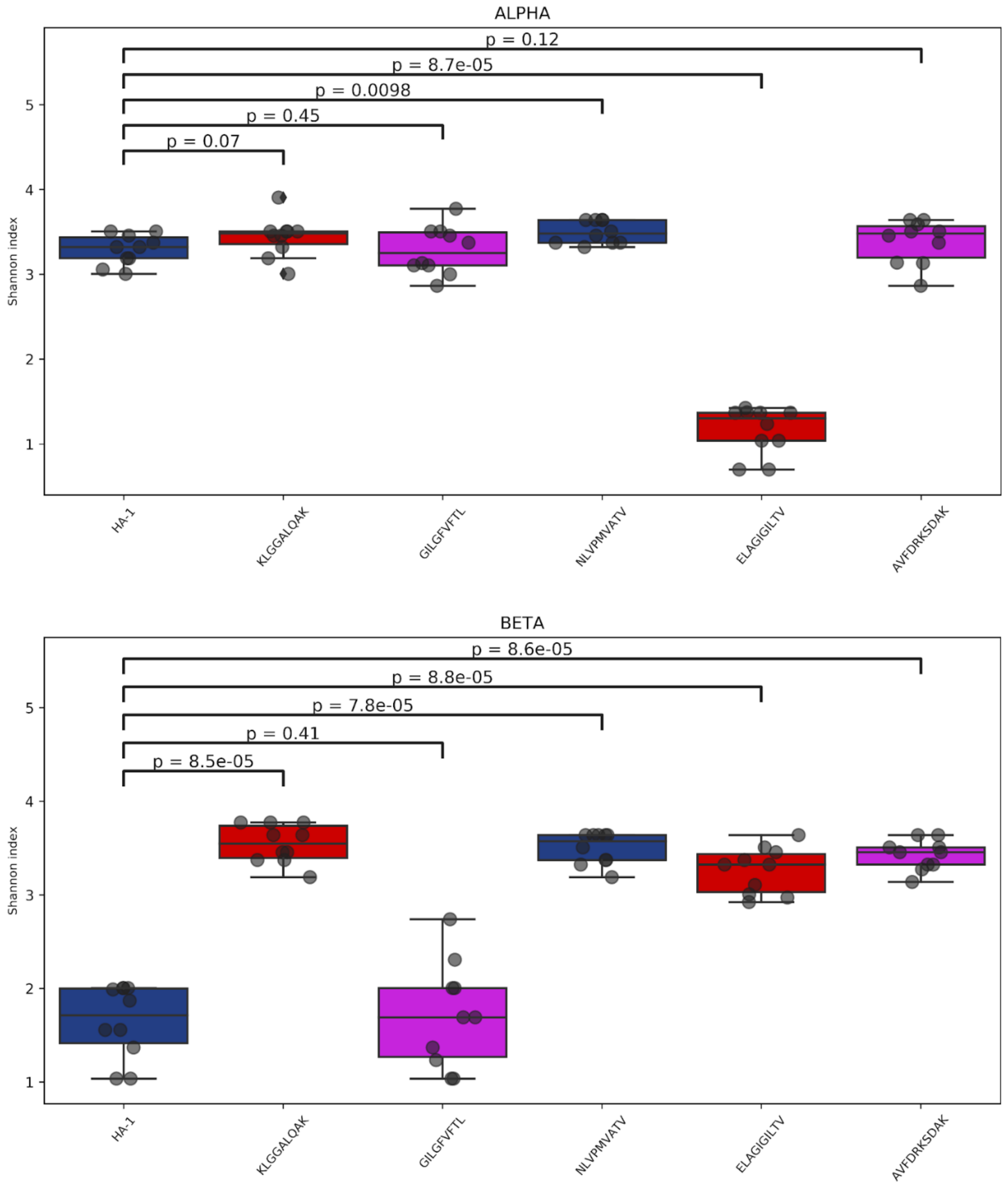

Supplementary Figure 3. **Shannon diversity index of TRAV and TRBV genes of HA-1-specific TCR.** The repertoire was compared to the repertoires with other specificities: KLG (Cytomegalovirus), GIL (Influenza A), NLV (Cytomegalovirus), ELA (MLANA protein, *Homo Sapiens*), AVF (Epstein-Barr virus). Data sets were obtained from VDJdb (https://vdjdb.cdr3.net). The HA-1-specific TCRs were randomly divided into 9 sets, the Shannon index was calculated for each set separately and plotted on the graph along with calculated p-value (Student t-test).

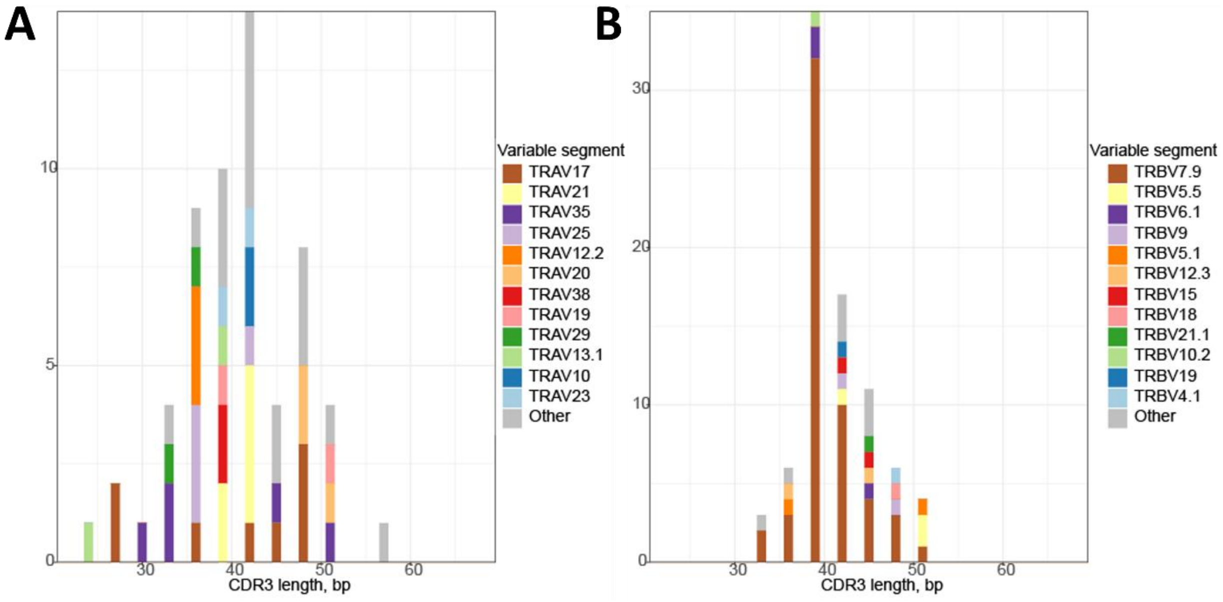

Supplementary figure 4. **CDR3 length distribution for HA-1 specific TCR**. Lengths of α (A) and β (B) chains were determined from the NGS sequencing data for HA-1 repertoire. Share of each V gene in the TCRs with the specific CDR3 length is indicated by color.

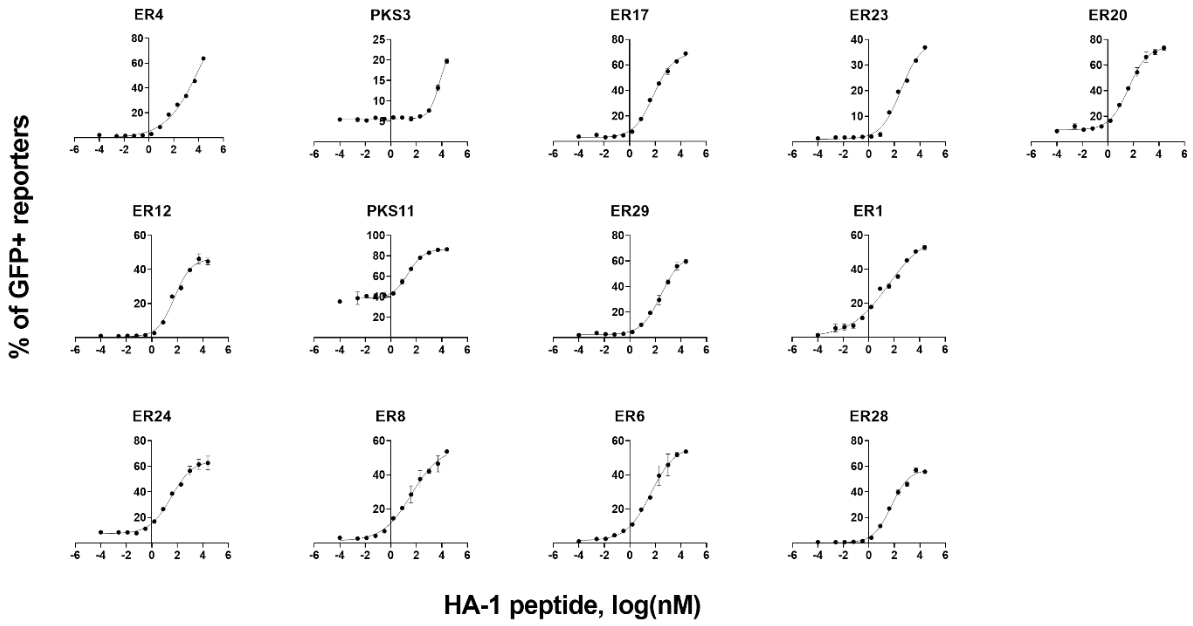

Supplementary figure 5. Representative titration curves for TCR affinity estimation. HA-1 peptide titration curves for HA-1-specific TCR receptors plotted as percentage of the GFP^{+} reporter cells according flow cytometry. Peptide titration assay was performed using transgenic J76 cells expressing indicated TCR receptors and HA-1 presenting K562 cell line with transgenic HLA-A*02 allele pulsed with five-fold dilutions of exogenous peptide. HA-1. Results of one representative experiment in two replicates are shown for each analysed TCR construct, error bars represent the mean absolute errors.

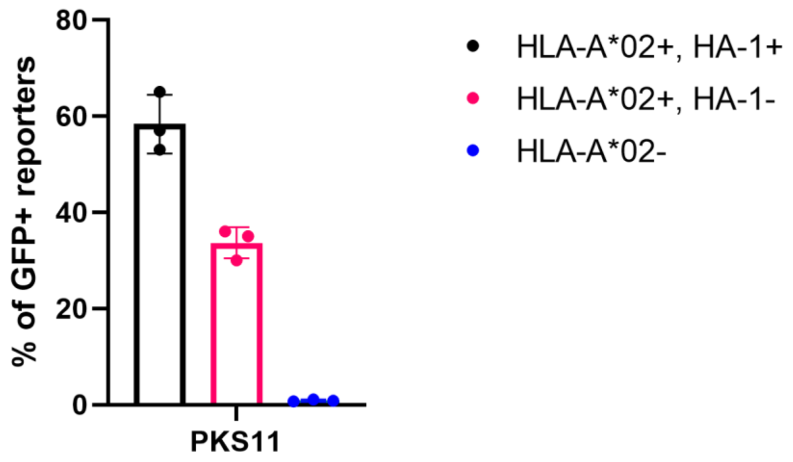

Supplementary figure 6. TCR PKS11 demonstrates alloreactivity on a set of healthy donor PBMC. Stimulation of the J76 reporter cells with expressing PKS11 TCR receptor by healthy donor PBMCs with different HA-1/HLA-A*02 genotypes. Responses were measured as percentage of GFP^+ reporter cells by flow cytometry. Stimulation was performed using PBMC of three donors for each group. Error bars represent the mean absolute error.

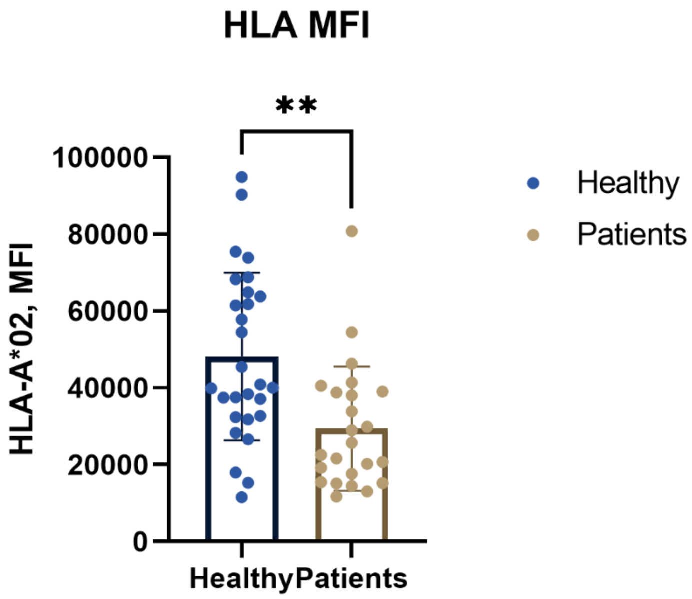

Supplementary figure 7. **Patient PBMC demonstrate lower lever of HLA staining than PBMC from healthy donors.** Mean HLA-A*02 surface expression on the cells from PBMC of healthy donors and leukemia patients measured as MFI of the HLA-A*02 staining by flow cytometry. Statistical significance: unpaired t-test **p=0.001

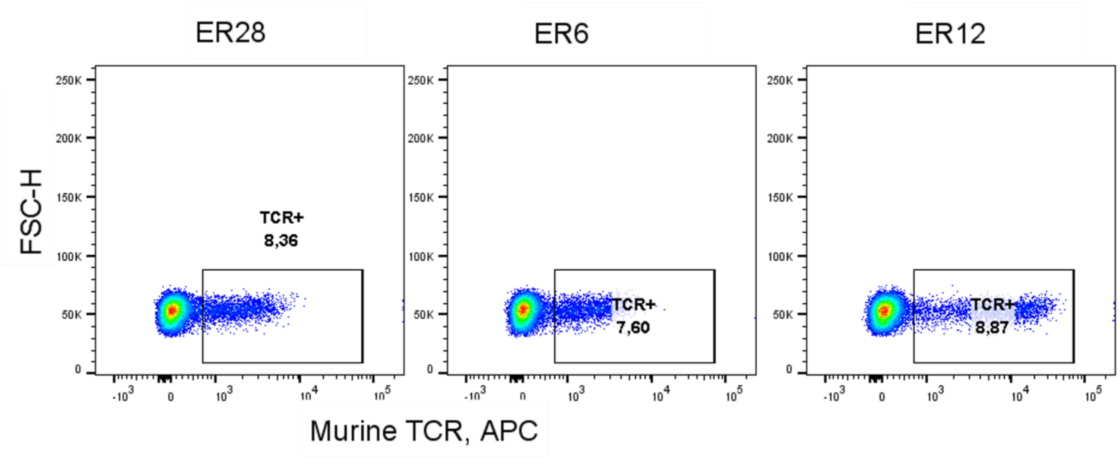

Supplementary figure 8. Parallel CD8$^+$ T cell transductions show consistent efficiency. Expression of murinised TCRs ER6, ER12 and ER28 in the CD8$^+$ T cells stained with murine TCR-APC conjugates (2133590, Sony, Japan) 72h after transduction and measured by flow cytometry.

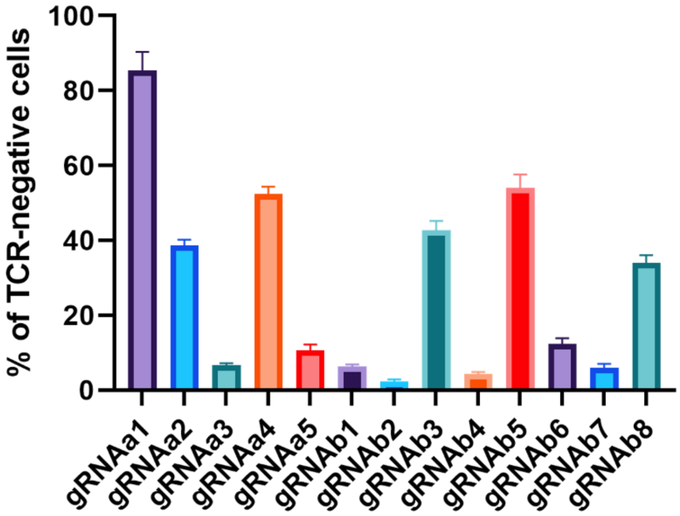

Supplementary figure 9. TCR knockout efficiency significantly varies for different gRNAs. Efficiency of TCR knockout by different gRNAs after RNP electroporation of Jurkat E6-1 cells. Cells were stained with anti TCR antibodies 72 h after electroporation and the percentage of TCR-negative cells was determined by flow cytometry according to *supplementary materials*. Error bar represents the mean error, n=3.

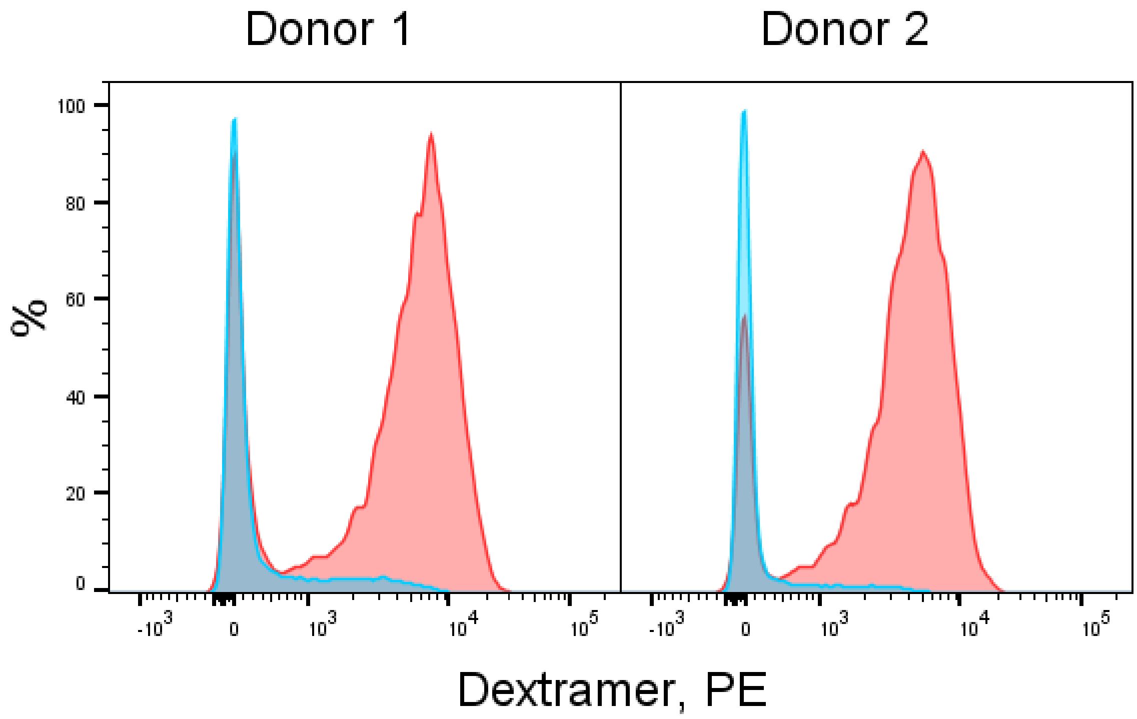

Supplementary figure 10. Enriched gene-modified CD8$^+$ cultures from two donors retain specificity to HA-1 after expansion. HA-1 HLA-A*02.01 dextramer staining of non-enriched (blue) and enriched by immunomagnetic separation transgenic HA-1-specific CD8$^+$ T cells expressing murinised TCR (red). The enriched cells from 2 donors retain up to 90% of antigen-specific cells after expansion. Expansion of both enriched and non-enriched cultures was for 14 days.

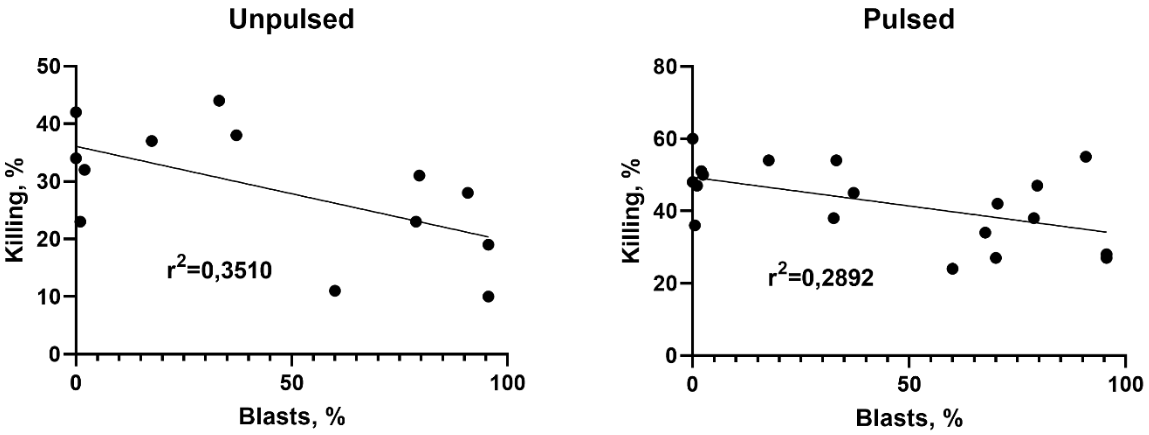

Supplementary figure 11. Cytotoxic effect of the HA-1-specific transgenic T cells is not significantly correlated with the blast percentage in the PBMC samples. Linear regression method was applied for analysis. Each dot corresponds to the killing efficiency of transgenic $CD8^+$ T cells observed for each patient sample with indicated blast percentage, Correlation data were calculated as mean between two independent experiments with different donors of used effector cells (data from supplementary table 4).
